# Burnout, quality of life, motivation, and academic achievement among medical students: A person-oriented approach

**DOI:** 10.1007/s40037-017-0340-6

**Published:** 2017-02-28

**Authors:** Mataroria P. Lyndon, Marcus A. Henning, Hussain Alyami, Sanjeev Krishna, Irene Zeng, Tzu-Chieh Yu, Andrew G. Hill

**Affiliations:** 1 Ko Awatea, Counties Manukau Health, Auckland, New Zealand; 2 Department of Surgery, South Auckland Clinical Campus, The University of Auckland, Auckland, New Zealand; 3 Centre for Medical & Health Sciences Education, The University of Auckland, Auckland, New Zealand

**Keywords:** Medical education, Progress testing, Burnout, Academic motivation, Quality of life

## Abstract

**Background:**

The aim of this study was to identify burnout and quality of life profiles of medical students and determine their associations with academic motivation and achievement on progress tests using a person-oriented approach.

**Methods:**

Medical students (*n* = 670) in Year 3 to Year 5 at the University of Auckland were classified into three different profiles as derived from a two-step cluster analysis using World Health Organization Quality of Life-BREF scores and Copenhagen Burnout Inventory scores. The profiles were used as independent variables to assess differences in academic motivation and achievement on progress tests using a multivariate analysis of co-variance and repeated measures analysis of co-variance methods.

**Results:**

The response rate was 47%. Three clusters were obtained: Higher Burnout Lower Quality of Life (*n* = 62, 20%), Moderate Burnout Moderate Quality of Life (*n* = 131, 41%), and Lower Burnout Higher Quality of Life (*n* = 124, 39%). After controlling for gender and year level, Higher Burnout Lower Quality of Life students had significantly higher test anxiety (*p* < 0.0001) and amotivation scores (*p* < 0.0001); and lower intrinsic motivation (*p* < 0.005), self-efficacy (*p* < 0.001), and progress test scores (*p* = 0.03) compared with the other profiles.

**Conclusion:**

Burnout and Quality of Life profiles of medical students are associated with differences in academic motivation and achievement over time.

## What this paper adds

- A person-oriented approach can identify specific burnout and quality of life profiles among medical students. By using a person-oriented approach this study has demonstrated an important relationship between burnout and quality of life profiles, academic motivation and achievement on progress tests over time. Understanding burnout and quality of life profiles could assist in customizing support activities for students within each profile.
- This is the first study in medical education that classifies students according to burnout and quality of life profiles using a person-oriented approach. It presents opportunities for future research and elaboration on study findings.

## Introduction

There is growing concern about the well-being of medical students during medical school [1–3]. This is not surprising given the high rates of burnout and poor quality of life reported among medical students. Indeed, a study involving 1098 medical students in the US found that 45% of their sample met the criteria for burnout, and scored lower on mental quality of life scores when compared with the general population [4]. These findings are consistent with other studies noting that medical students are vulnerable to psychological distress that may impact on their academic achievement, and motivation [1, 5].

Previous studies in the medical education literature have investigated the relationships between psychological distress, motivation, and academic performance [5–8]. Artino et al. [6] reported that task value and self-efficacy motivation is positively correlated with student enjoyment and course examination grade, while anxiety is negatively correlated with course examination grade. Similarly, a study by Park et al. [8] showed an association between stress, academic motivation and achievement. In this study, students with higher stress scores scored higher on amotivation and lower on their grade point average than students with lower stress scores.

Henning et al. [5] investigated associations between medical students’ perceptions of quality of life, motivation to learn and self-disclosed academic achievement. The findings of their study suggested positive correlations between quality of life, motivation to learn and written examination grades. However, Del-Ben et al. [7] found that increased anxiety, decreased academic motivation and a maladjusted leisure and social life had no significant correlations with examination grades.

These studies have explored the relationship between psychological distress or quality of life with study outcomes as group variables, which is known as a variable-oriented approach [9]. This approach is useful for understanding how psychological distress and quality of life influences motivation and academic achievement, and also the direction of influence.

However, an alternative approach is to look at how individual students differ in their levels of psychological distress and quality of life and how this relates to their academic outcomes. In this ‘person-oriented approach’, the individual student is seen as a functioning totality, best studied by analyzing patterns of information together, not separate and isolated variables as is the case in a variable oriented approach [10].

Within the context of quality of life and burnout, a person-oriented approach could firstly promote understanding of students as unique individuals by considering the many facets of quality of life and burnout experienced within a student rather than considering each facet in isolation [9, 11]. Secondly, a person-oriented approach can categorize individual students into distinct profiles whose members share similar burnout and quality of life characteristics. From a theoretical perspective, this approach can offer novel and unique insights about inter-individual differences and intra-individual variation that could be overlooked or misunderstood in variable-oriented approaches [12]. From a practical perspective, such information might be useful both from a diagnostic viewpoint and from an intervention viewpoint [13]. For instance, interventions to address burnout and enhance quality of life can be tailored to each particular profile.

Although a person-oriented approach presents theoretical and practical opportunities, no studies in medical education have utilized a person-oriented approach to exploring burnout and quality of life, and their effects on academic motivation and achievement. Therefore, the objective of this study was to utilize a person-oriented approach to identify burnout and quality of life profiles of medical students, and determine their associations with academic motivation and achievement.

## Method

### Subjects and procedures

The participants in this study were self-selected volunteers in Year3, Year 4 and Year 5 of a six year undergraduate program at the University of Auckland, New Zealand. The first part of this study was carried out over a two-week period between June and July 2014. At the end of a lecture, students were given a self-report Likert-type questionnaire composed of validated measures of academic motivation, burnout, and quality of life. Contained within the questionnaire was the Academic Motivation Scale, which measured intrinsic motivation, extrinsic motivation and amotivation, and subscales of the Motivated Strategies for Learning Questionnaire (MSLQ), which measured self-efficacy and test anxiety [14, 15]. The Academic Motivation Scale and MSLQ have been previously used in medical education research, both internationally and in New Zealand, and among a University of Auckland medical student population [5, 16, 17].

Consistent with studies by Kusurkar et al. [9], the author modified the Academic Motivation Scale, which was originally designed for college and university students, so that it could be applied to medical students and further checked the reliability of each scale. Intrinsic motivation scores were calculated from the Academic Motivation Scale as an average of the intrinsic motivation scores on the three subscales [9]. Extrinsic motivation scores were calculated by taking an average of introjected regulation and external regulation scores [9]. The identified regulation subscale was not included within calculations and subsequent data analysis as the items on this subscale are such that most students in professional education would answer positively [9]. Therefore, this subscale is not likely to discriminate within a medical student population [18].

The questionnaire also contained a World Health Organization quality of life questionnaire (WHOQOL-BREF), and the ‘personal burnout’ scale from the Copenhagen Burnout Inventory to measure quality of life and burnout respectively [19, 20]. Both the WHOQOL-BREF and the Copenhagen Burnout Inventory have been used in a number of quality of life and burnout studies during medical training, and the WHOQOL-BREF has been previously used among the University of Auckland medical student population [5, 21, 22]. The study questionnaire also contained a survey of student characteristics (including age, gender, admission scheme into medical school, and year level of the curriculum).

The WHOQOL-BREF questionnaire included 24 items that encompass four quality of life domains (physical health, psychological health, social relationships and environmental conditions). The scores for each domain were calculated using a well-recognized WHOQOL-BREF syntax [23]. The personal burnout subscale of the Copenhagen Burnout Inventory contains six items with scoring from 0–100 for each item. The total score on the scale was the mean of the scores on the items [20].

The second part of this study was carried out in October 2014, which included collecting student academic performance data based on progress tests completed in April, July and October 2014 respectively.

### Ethics approval and statistical analyses

Written informed consent to participate was obtained from all students, and ethics approval was obtained from the University of Auckland Human Participants Ethics Committee (UAHPEC Ref 8467).

All statistical analyses were performed using IBM SPSS 22.0 for Mac. Internal reliability measures, Cronbach’s alpha coefficients, for each section of the questionnaires were determined.

Participants were classified to different profiles based on WHOQOL-BREF and Copenhagen Burnout Inventory scores using a two-step cluster analysis [24]. The two-step clustering method within the auto-cluster modelling node was chosen because of its ability to handle both continuous and categorical variables. The two-step cluster analysis method operates through firstly scanning the data in a pre-classificatory stage and identifying ‘dense’ regions of data that share similar values across a range of variables [25]. An algorithm similar to an agglomerative hierarchical clustering method is then used to classify the data [24]. The algorithm used the log-likelihood distance measure and Schwarz’s Bayesian Criterion to derive the cluster solutions (burnout and quality of life profiles) by maximizing between-group heterogeneity and within-group homogeneity, thereby capturing the interactions between dimensions of quality of life and burnout.

Once the burnout and quality of life profiles were derived, Chi-square analyses were conducted to determine any significant differences in characteristics between profiles. Any significant differences were then included in a multivariate analysis of covariance (MANCOVA) model as covariates.

A MANCOVA model was used to determine differences between profiles in relation to academic motivation, self-efficacy and academic performance. Profile membership was included in the model as the independent variable, and the dependent variables were intrinsic motivation, extrinsic motivation, amotivation, test anxiety and self-efficacy scores A correlation matrix that presents the correlations between the dependent variables in the MANCOVA model is included in Table 1. Post hoc multiple group comparisons were adjusted using the Bonferroni correction for controlling Type 1 error. The effect size was calculated from partial eta squared: small = 0.0–0.06, medium = 0.06–0.138, large >0.138 [26].

**Table 1:** Correlation matrix between the response variables in the MANCOVA model

	Intrinsic motivation	Extrinsic motivation	Amotivation	Self-efficacy	Test anxiety
Intrinsic motivation	1	0.41**	−0.26**	0.31**	−0.04
*N*	322	322	322	321	320
Extrinsic motivation	0.41**	1	0.14*	0.20**	0.07
*N*	322	322	322	321	320
Amotivation	−0.26**	0.14*	1	−0.21**	0.10
*N*	322	322	322	321	320
Self-efficacy	0.31**	0.20**	−0.21**	1	−0.28**
*N*	321	321	321	321	320
Test anxiety	−0.04	0.07	0.10	−0.28**	1
*N*	320	320	320	320	320

*Correlation is significant at the 0.05 level (2-tailed)

**Correlation is significant at the 0.01 level (2-tailed)

A separate repeated measures analysis of covariance (ANCOVA) method was also used to compare changes in academic achievement over time. Profile membership was included in the model as the independent variable, and the three progress test scores were designated as dependent variables. Year level and gender were included in both models as potential confounding variables.

Pearson’s correlations were also used to determine any associations between quality of life domain scores, and academic motivation scores.

## Results

The response rate was 47%. Of these respondents, 44% were male and 56% were female. The two-step cluster analysis derived three distinct cluster solutions (profiles) with a silhouette coefficient of 0.3 which represents a fair cluster solution [25].

The frequencies and proportions of students represented in each of the burnout and quality of life profiles are shown in Table 2. Thirty-nine percent of students were represented in the Lower Burnout Higher Quality of Life profile, 41% of students were in the Moderate Burnout Moderate Quality of Life and the remaining 20% were in the Higher Burnout Lower Quality of Life.

**Table 2:** Demographics and characteristics of profiles

Profile membership *N* (%)	Higher Burnout Lower QOL (HBLQ)	Moderate Burnout Moderate QOL (MBMQ)	Lower Burnout Higher QOL (LBHQ)	Total
No. of students	62 (20%)	131 (41%)	124 (39%)	316
Males	21 (15%)	54 (39%)	64 (46%)	139
Females	40 (23%)	77 (44%)	60 (33%)	177
Age (mean)	22.9	22.8	24.4	–
Year 3	18 (21%)	44 (50%)	26 (29%)	88
Year 4	18 (20%)	41 (46%)	31 (34%)	90
Year 5	26 (19%)	46 (33%)	67 (48%)	139

The results of the chi square analysis showed the distribution of gender (χ^2^ (2, *n* = 316) = 5.60, *p* = 0.061) and admission status (χ^2^ (6, *n* = 315) = 3.95 *p* = 0.684) were not significantly different between profiles. However, student year level was significantly different between profiles (χ^2^ (4, *n* = 317) = 9.86 *p* = 0.043). A higher proportion of students in Year 3 of the medical program were in the Higher Burnout Lower Quality of Life and a lower proportion were in the Lower Burnout Higher Quality of Life profiles compared with other year levels (Table 2). Therefore, year level was controlled for while conducting subsequent analyses. Gender was also controlled for in subsequent analyses as it was nominally significant (*p* = 0.061) and previous literature has suggested a gender effect in relation to psychological distress [2].

All measures were found to be internally consistent and within acceptable limits [27, 28]. Correlation analysis showed that burnout was positively correlated with amotivation (*r* = 0.26, *p* < 0.001) and test anxiety (*r* = 0.36, *p* < 0.001), and negatively correlated with intrinsic motivation (*r* = −0.16, *p* = 0.005), and self-efficacy (*r* = −0.29, *p* < 0.001) Furthermore, dimensions of quality of life were positively correlated with intrinsic motivation, and self-efficacy, and negatively correlated with amotivation and test anxiety (Table 3).

**Table 3:** Correlations between burnout and quality of life with academic motivation

	Intrinsic motivation	Extrinsic motivation	Amotivation	Self-efficacy	Test anxiety
CBI-MEAN	−0.16**	0.01	0.26**	−0.23**	0.36**
Physical QOL	0.17**	−0.02	−0.17**	0.28**	−0.24**
Psychological QOL	0.20**	−0.01	−0.31**	0.35**	−0.33**
Social QOL	0.08	−0.08	−0.31**	0.18**	−0.15**
Environmental QOL	0.11*	0.06	−0.12	0.20**	−0.24**

*CBI* Copenhagen Burnout Inventory

**Correlation significant at the 0.01 level (2-tailed)

*Correlation significant at the 0.05 level (2-tailed)

In relation to burnout and quality of life profiles, the results of the MANCOVA indicated significant differences in academic motivation for different profiles, with Higher Burnout Lower Quality of Life scoring lower on intrinsic motivation and self-efficacy, and higher on amotivation and test anxiety (Table 4). The largest effects tended to be associated with test anxiety and amotivation, which had effect sizes of medium to large [26].

**Table 4:** Academic motivation least square mean scores (LSM) and standard errors (SE) by profile membership

Profile membership LSM (SE)*	Higher Burnout Lower QOL	Moderate Burnout Moderate QOL	Lower Burnout Higher QOL	*P* value**	Effect size –partial Eta squared^c^
Intrinsic^a^	3.25 (0.08)	3.46 (0.06)	3.57 (0.06)	0.006	0.03
Extrinsic^a^	2.90 (0.11)	3.00 (0.08)	2.82 (0.08)	0.240	0.01
Self-efficacy^b^	3.27 (0.08)	3.52 (0.05)	3.64 (0.06)	0.001	0.04
Test anxiety^b^	3.52 (0.12)	2.86 (0.08)	2.54 (0.08)	<0.0001	0.13
Amotivation^a^	1.64 (0.08)	1.36 (0.05)	1.18 (0.05)	<0.0001	0.07

*The reporting least square means (LSMs) are adjusted for gender and year level

** *P* values from MANCOVA

^a^Measured by a modified version of the AMS; range of scores = 1 to 5

^b^Measured by the MSLQ; range of scores = 1 to 5

^c^Effect sizes from partial Eta squared: Small = 0.01–0.06, Medium = 0.06–0.138, Large >0.138 [23]

Post hoc mean comparisons were statistically significant (*p* < 0.05) (Table 5). The Higher Burnout Lower Quality of Life students had higher amotivation and test anxiety and lower intrinsic motivation and self-efficacy compared with the other profiles. For the Higher Burnout Lower Quality of Life compared with Moderate Burnout Moderate Quality of Life and Lower Burnout Higher Quality of Life, the mean differences in intrinsic motivation scores were −0.3 (0.1) and –0.3 (0.1), differences in amotivation scores were 0.3 (0.1) and 0.5 (0.01), differences in self-efficacy were −0.3 (0.1) and −0.4 (0.1), and differences in test anxiety were 1.0 (0.1) and 1.0 (0.1) respectively.

**Table 5:** Post hoc comparisons of burnout and quality of life between profiles only, including significant dimensions of the domain

Academic motivation	Comparisons between profiles	Mean difference*	Standard error	*P* value^a^	95% CI for difference^a^
Intrinsic motivation	HBLQ vs LBHQ	−0.31	0.10	0.004	(−0.56,−0.08)
Amotivation	HBLQ vs MBMQ HBLQ vs LBHQ	0.28 0.46	0.09 0.95	0.008 <0.001	(0.05, 0.51) (0.24, 0.69)
Self-efficacy	HBLQ vs MBMQ HBLQ vs LBHQ	−0.25 −0.37	0.10 0.08	0.027 0.001	(−0.48,−0.02) (−0.60,−0.13)
Test anxiety	HBLQ vs MBMQ HBLQ vs LBHQ	0.66 0.98	0.14 0.14	<0.001 <0.001	(0.32, 1.00) (0.63, 1.32)

*CI* confidence interval, *HBLQ* Higher Burnout Lower QOL, *MBMQ* Moderate Burnout Moderate QOL, *LBHQ* Lower Burnout Higher QOL

*The mean difference is significant at the 0.05 level

^a^Adjustment for multiple comparisons: Bonferroni

**Fig. 1:**
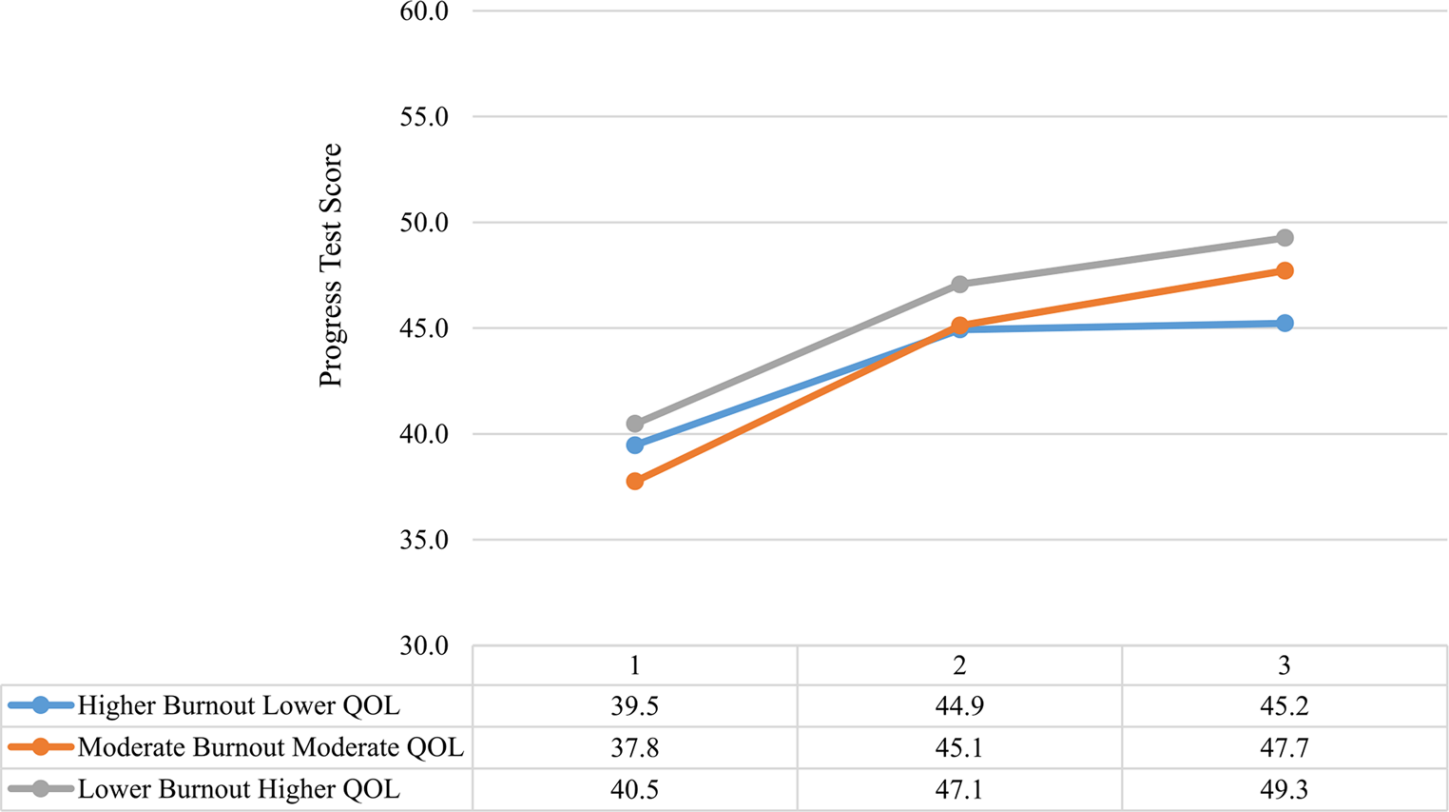
Progress test scores for each profile over time

In reference to the differences in progress test scores, the results of the repeated measures ANCOVA showed a significant time effect between profiles (*p* = 0.029) (Fig. 1). The Higher Burnout Lower Quality of Life students scored significantly lower on the third (end of year) progress test when compared with the Lower Burnout Higher Quality of Life and Moderate Burnout Moderate Quality of Life profiles. The magnitude of this effect was small.

## Discussion

The aim of this study was to identify burnout and quality of life profiles of medical students and determine their associations with academic motivation and achievement. By taking a person-oriented approach, this study found that medical students display specific profiles of burnout and quality of life that are associated with differences in academic motivation and progress test scores over time.

The findings in this study showed that students in their early stages of medical education were over-represented in the Higher Burnout Lower Quality of Life profile. The students in this profile had the least desirable type of academic motivation, with lower intrinsic motivation and self-efficacy, higher amotivation and higher test anxiety, and lower scores on progress tests over time. In comparison, students in the Lower Burnout Higher Quality of Life profile had a more optimal motivational orientation, with higher intrinsic motivation and self-efficacy, and lower test anxiety and amotivation. These findings are consistent with other studies in the medical education literature in which differences in burnout and quality of life between year levels of the medical curriculum have been found [29, 30]. These findings are also consistent with the study by Park et al. [8], who reported that psychological distress positively correlates with amotivation and negatively correlates with intrinsic motivation and achievement. Similarly, Henning et al. [5] reported that poorer quality of life is associated with lower achievement and positively correlated with test anxiety. Thus, the present study presents further evidence of the effects of psychological distress and poor quality of life on academic motivation and achievement among specific subgroups of medial students.

By identifying specific burnout and quality of life profiles, a person-oriented approach yielded complementary information to studying burnout and quality of life using a variable-oriented approach. The present study builds on previous variable-oriented research by providing an insight into the many facets of quality of life and burnout experienced by students. As this study has shown burnout and quality of life to be integrally linked, it seems logical from both a research and a pastoral care perspective to consider them together through a person-oriented approach, rather than in isolation. Therefore, the implications of this approach may be customizing pastoral support activities to employ a broader repertoire of wellness promotion practices to address, not only burnout, but also the wider quality of life issues that students often face.

## Directions for future person-oriented research

This is the first study in medical education that classifies students according to burnout and quality of life profiles using a person-oriented approach. As it is the first of its kind, it presents opportunities for future research and elaboration on study findings. For example, this study was exploratory and therefore the identification of burnout and quality of life profiles were seldom theoretically derived, but were derived statistically a using cluster analysis. Future studies could be more theory driven in identifying burnout and quality of life profiles and interpreting results. This may lend the opportunity to explain the nature of the interactions between burnout and quality of life within each profile as well as interactions with other factors such as gender or curriculum variables.

Secondly, as these data are cross-sectional, we cannot confirm whether these associations would change over time. It would be beneficial as part of a future research agenda to investigate whether these profiles remain stable during medical study or change according to the learning environment and experience. A longitudinal study design to study these aspects would be ideal.

Third, this study was conducted at a single academic institution, and therefore may not be generalisable to the wider medical student population. A multi-centre study could improve generalisability.

## Conclusion

By using a person-oriented approach this study has demonstrated an important relationship between burnout and quality of life profiles, academic motivation and achievement on progress tests over time. Lower Burnout Higher Quality of Life students had more optimal academic motivation with higher intrinsic motivation and self-efficacy and lower amotivation and test anxiety. Higher Burnout Lower Quality of Life students had the least desirable type of academic motivation, with lower intrinsic motivation and self-efficacy, and higher amotivation and test anxiety. Students in this profile also had lower academic achievement on progress tests over time.
